# 苯达莫司汀联合托法替布治疗T幼淋巴细胞白血病1例

**DOI:** 10.3760/cma.j.cn121090-20231024-00227

**Published:** 2024-04

**Authors:** 银芬 邓, 小丽 钱, 月 许, 筱 杨, 红建 袁

**Affiliations:** 扬州大学附属泰州第二人民医院血液科，泰州 225500 Department of Hematology, the Affiliated Taizhou Second People's Hospital of Yangzhou University, Taizhou 225500, China

患者，男，66岁，2022年11月3日因“胸闷、气急5 d”就诊于我院，门诊查血常规：WBC 230.75×10^9^/L、淋巴细胞计数216.67×10^9^/L、单核细胞计数6.69×10^9^/L、ANC 6.92×10^9^/L、RBC 4.00×10^12^/L、HGB 130 g/L、PLT 78×10^9^/L。胸部CT：胸腔左侧大量积液，左肺不张，纵隔右偏，双肺炎症改变，脾脏肿大。遂至血液科住院。

查体：神志清，精神一般，呼吸稍促，右侧颈部可触及3～4枚黄豆大小淋巴结，左侧颈部可触及1枚黄豆大小淋巴结，右侧腋窝可触及1枚蚕豆大小淋巴结，双侧腹股沟可触及2～3枚黄豆至蚕豆大小淋巴结，均质韧，无触痛，活动度一般。颈软，气管偏右，颈静脉无怒张。右肺呼吸音粗，下肺可闻及少许湿啰音，左上肺呼吸音减低，余肺呼吸音未闻及。脾脏肋下3 cm，质韧，无触痛，余无阳性体征。入院后查LDH 704 U/L，β_2_微球蛋白4.87 mg/L。肝肾功能、甲状腺功能、免疫、肝炎、结核、病毒指标未见异常。肿瘤指标：糖链抗原12 562.60 U/ml。血清蛋白电泳阴性。胸腔B超：左侧胸腔第4到11肋间可见液性暗区，最大深度约98 mm，距体表约12 mm。行左侧胸腔闭式引流，呈血性胸水，胸水中有核细胞数为168.46×10^9^/L，淋巴细胞比例为85％；胸水免疫分型：流式细胞术检测结果表明送检胸水标本中可见约95.13％异常T细胞，其免疫表型为CD52^+^、CD2^+^、CD3^+^、CD4^+^、CD5^+^、CD7^+^、CD8^−^、CD56^−^、CD34^−^、CD117^−^、HLA-DR^−^。外周血细胞分类：粒细胞占6％。单核细胞占3％。淋巴细胞占91％，计数明显增高，形态未见明显异常。血小板计数偏低。提示慢性淋巴增殖性疾病。骨髓涂片：①取材，涂片，染色良好；②骨髓增生活跃+；③粒系占19％，红系占5.5％，形态未见异常；④淋巴细胞占71％，其中成熟淋巴细胞占70％，形态未见明显异常；⑤全片未见巨核细胞，血小板少见。骨髓象示：成熟淋巴细胞肿瘤可能。骨髓免疫分型：流式细胞术检测结果表明送检标本中可见约92.00％异常T细胞，其免疫表型为CD2^+^、CD3^+^、CD4^+^、CD5^+^、CD7^+^、CD8^−^、TCRγδ^−^、CD56^−^、CD57^−^、CD16^−^、CD34^−^、CD117^−^、TdT^−^、CD45RA^+^少量、CD45RO^−^。淋系白血病中常见融合基因筛查：阴性。骨髓病理：骨髓有核细胞增生程度极度活跃（造血面积约90％）；粒/红比例略增高；粒系少见，以偏成熟阶段细胞为主，偏幼稚细胞散在少数；红系少见，以中晚幼红细胞为主；巨核细胞数量在正常范围，以分叶核为主，有的胞体小，分叶少；淋巴细胞散在多见；骨髓间质未见胶原纤维增生。免疫组化：CD34小血管（<2％+）、CD117（1％+）、MPO（30％+）、CD3（40％+）、CD79a（5％+）、CD10（6％+）、MUM-1（−）、Ki-67（<20％+）。结合免疫组化，考虑T细胞肿瘤（肿瘤细胞约占40％）。染色体核型：PHA刺激核型44~45,X,-Y,add(5)(p15),add(6)(q23)inv(9)(p12q13)c,del(11)(q23),-13,-14,add(16)(q13),-18,add(19)(q13),add(21)(p11.2), -22,+2～4mar[cp16]/46XYinv(9)(p12q13)c[4]。T细胞克隆性评估：TCR-β、TCR-γ重排阳性，TCR-δ重排阴性。人类T淋巴细胞-1型病毒抗体阴性。二代测序：有强临床意义的变异：JAK3基因p. M511I、p.R657Q和p.V674A错义突变，突变频率分别为26.0％、6.7％和2.3％。有潜在临床意义的变异：ATM基因p.R3008H错义突变，突交频率为42.1％。临床意义尚未明确的变异：JAK1基因p.F8051错义突变，突变频率为4.0％。最终患者诊断为T幼淋巴细胞白血病（T-PLL）。

初始时我们认为该病临床侵袭性较强，故而选择了强化疗方案，为减少肿瘤溶解综合征的风险，治疗前行白细胞单采，后给予拆分剂量的hyper-CVAD（环磷酰胺+长春新碱+多柔比星+地塞米松）方案化疗，化疗期间淋巴细胞计数下降不明显，化疗结束后3 d患者淋巴细胞复升，继而予苯达莫司汀90 mg·m^−2^·d^−1^×2 d，联合托法替布5 mg每日2次治疗，同时口服阿昔洛韦、复方新诺明预防感染。用药后患者淋巴细胞逐渐降至正常（图1），胸水逐步吸收（图2），浅表淋巴结及脾脏逐渐缩小，现已完成7个疗程的苯达莫司汀+托法替布治疗，血常规维持正常水平，无浅表淋巴结及脾肿大，无明显骨髓抑制、感染、肝肾功能损害等并发症，末次化疗前骨髓检查（2023年7月21日）流式细胞术仅见0.5％的残留肿瘤细胞。二代测序提示JAK3基因突变阴性。染色体核型：46,XY,inv(9)(p12q13)[20]，为体质性遗传多态性。目前继续口服托法替布维持治疗，疾病缓解中。

**图1:**
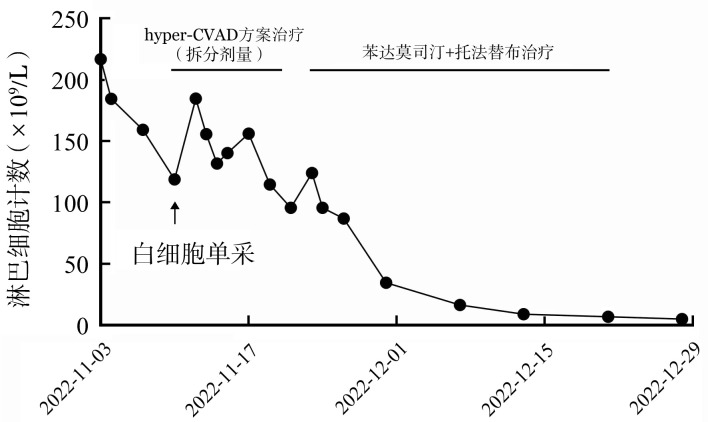
T幼淋巴细胞白血病患者治疗后淋巴细胞计数的变化趋势 **注** hyper-CVAD：环磷酰胺+长春新碱+多柔比星+地塞米松

**图2:**
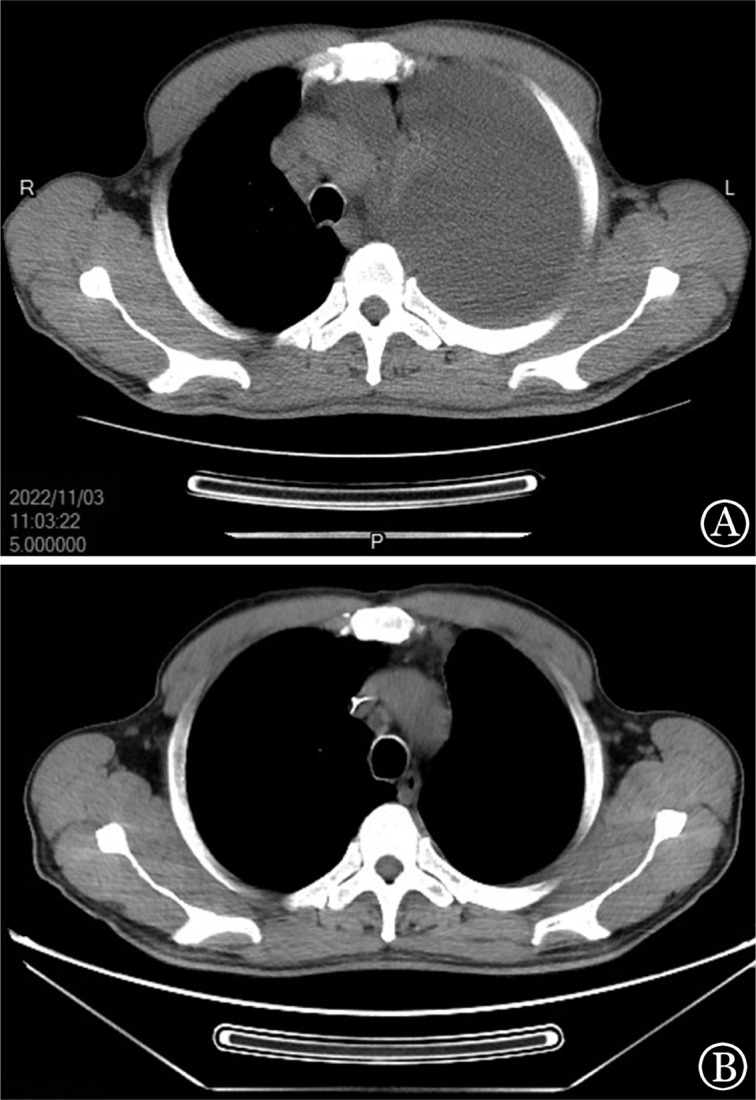
T幼淋巴细胞白血病患者治疗前后胸部CT的变化 **A** 治疗前（2022年11月3日）；**B** 苯达莫司汀+托法替布治疗后（2022年12月20日）

我们诊断了1例伴JAK基因突变的T-PLL，该患者对常规化疗不敏感，使用苯达莫司汀联合托法替布治疗后疾病缓解，提示苯达莫司汀联合JAK抑制剂治疗伴JAK基因突变的T-PLL是可行的选择。

